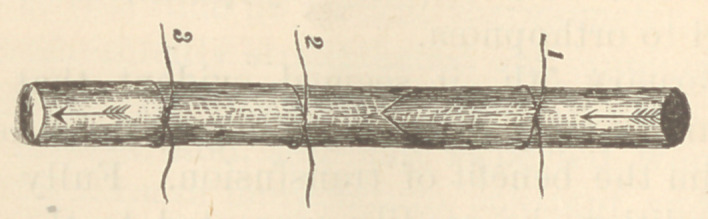# Transfusion of Defibrinated Human Blood, Etc.

**Published:** 1876-06

**Authors:** M. W. Wood

**Affiliations:** Ass’t. Surg. U. S. A.; Camp Sheridan, Neb.


### EXAMINER.

Vol. XXXIII. — JUNE, 1876. — No. 6.

### Original Communications.

### TRANSFUSION OF DEFIBRINATED HUMAN BLOOD IN A CASE OF TUBERCULAR PHTHISIS.

By M. W. WOOD, Asst. Surgeon, U. S. A.

(Read before the Chicago Society of Physicians and Surgeons.)

Although transfusion was known to the ancients, even before the commencement of the Christian era, reports of cases in which this operation has been successfully performed have not been so frequent as to render the subject a trite one, and the following account is submitted, as it may prove of interest to some member of the Society.

Private F— D—, Co. "K," 9th U. S. Infantry, age 27, occupation, previous to enlistment, tinsmith, and whose family history is free from deaths from consumption or allied causes, was admitted into the hospital at this post, Aug. 31st, 1875, as a case of anæmia, and until Nov. 1st the case was so regarded, and accordingly treated by remedies addressed to the hæmatosic function of the body.

At about the latter date, the case came under my charge, and as I suspected the deposit of tubercle, I

made frequent and very careful examinations to detect evidence of its presence, but with a negative result. This patient was also carefully examined at about the same time by two other physicians, neither of whom was able to discover any evidence of a tubercular deposit, although we were all sufficiently satisfied that, sooner or later, tuberculosis would terminate the history of the case. I had at this time no clinical thermometer, which would have undoubtedly strengthened our opinions as to the correctness of the diagnosis. About Nov. 15th, my thermometer arrived, and, as I had expected would be the case, an elevation of temperature, constant, and from 1° F. to 2.5° F., was observed.

About the middle of December, I was hurriedly summoned one evening to the first hæmoptysis, which had, however, been preceded for twenty-four hours by bloody-streaked sputa, but without any marked fluctuation of temperature. From this time onward, despite the assiduous use of every means, therapeutic and hygienic, at my command, he continued to have, every few days, hæmoptyses, varying in quantity from two to eight ounces. These were sometimes preceded by prodromic symptoms, but prophylactic measures seemed unavailing. He had, as his constant companion, a small quantity of finely powdered table salt, with which he was able to arrest the flow of blood in a very short time, and he soon lost all fear of an immediate fatal result. During a period of two weeks he took one fluid drachm of aromatic sulphuric acid daily, but during this period hæmoptyses occurred as frequently and as profusely as before. Dilute phosphoric acid, tannic and gallic acids, and the tincture of the chloride of iron, successively thoroughly tried in full doses, were no more efficacious, and finally it was determined to distress the stomach no longer with astringents, reserving it for nutrition. Hæmoptyses were now no more frequent than before, but these, with the very rapidly advancing, and now very evident tuberculosis, were fast drawing the curtain upon a scene of patient suffering.

He had taken to his bed at last, but little more than a shadow, too weak to walk or stand alone ; his appetite had disappeared, and although he fully appreciated the importance of taking nutrition, barely two ounces of milk were taken daily, in addition to the cod-liver oil, of which he has taken about two gallons. He had for some time suffered from dyspnœa, and this now amounted to orthopnœa.

On February 5th, it seemed evident that he could hardly survive forty-eight hours longer, and I determined to give him the benefit of transfusion. Fully conscious of his condition, he readily consented to the operation as offering a hope ; worse he could not well be, and live. A young, strong, healthy man having volunteered as donor, a sufficient quantity of blood was drawn, and with an ordinary hard rubber syringe, between three and four ounces were injected, and even this small quantity proved sufficient to overcome the "dead point" as Dalton has happily termed it.

The apparatus used, and the mode of procedure adopted were so simple that I cannot forbear entering into details concerning them. Upon a retort-stand or funnel-holder, and over a spirit-lamp, a small tin basin, partly filled with water, was supported ; and in this, separated from its bottom by a small ring of pasteboard, was placed a wedgewood mortar which had been previously measured and graduated to indicate the quantity of blood drawn. The stem and attached scale of an ordinary thermometer were then placed in the bath so that at a glance the temperature might be read. The mortar was first heated to 105° F., and into this seven ounces of *venous* blood were drawn ; after which the apparatus was suffered to cool to 100° F., at which temperature it was kept thereafter, during the operation. The blood was carefully defibrinated by a wisp cut from a broom, and afterward constantly stirred, that its temperature might be uniform throughout.

The left median cephalic vein of the patient was chosen, and exposed for about an inch of its course, by dividing with scissors a transverse fold of skin pinched up between the fingers. About the vein thus exposed, three ligatures were thrown.

As this operation has not, to my knowledge, previously been performed in this manner, I will append a rude diagram.

I will speak of these ligatures as Nos. 1, 2, and 3. No. 1, as will be seen, is toward the distal extremity of the vein, and intended solely to prevent loss of blood by the patient; No. 3, toward the proximal extremity, to guard against the introduction of air; No. 2, nearer 3 than 1, to embrace the contained nozzle of the syringe during the injection. A valvular opening was then made into the vein with scissors, as indicated. The syringe was now filled with blood, its point introduced into the vein nearly to No. 3, and enough of its contents forced out to deprive this portion of vein of any air which it might contain. No. 2 was then tightened about the nozzle, No. 3 loosed, and the syringe emptied. No. 3 was now tightened, No. 2 loosed, and the syringe withdrawn. This process was repeated until nearly four ounces of blood had been injected.

The condition of the patient may be inferred from the brief account of his case already given; suffice it to say, that at 12 M., just before the operation, his temperature was 102.6° F.; pulse, 105; respirations, 34; orthopnœic and in great distress. At 1 P. M., after the operation, these were: temperature, 101.3° F.; pulse, 101; respirations, 27; and now he was breathing much easier; felt quite comfortable, and his lips, ears and nose had assumed a healthier hue. At 4.30 P. M., temperature, 104 F.; pulse, 111; respirations, 22, and he was much easier than he had been before for some days. From that hour until the present he has continued steadily to improve, and the minute details are unnecessary. The last time that he was weighed prior to the operation (some time in January) he weighed 97 lbs. ; and at the date of writing this, Feb. 25, 1876, he weighed 111½ lbs. This tells the story much better than can words of mine.

The night sweats, which had resisted all measures for their relief, disappeared the third night after the transfusion, and there has been no further hæmoptysis. The appetite has improved, and he now takes with relish, good nutritious food.

The dyspnœa is insignificant, and the destruction of lung tissue seems to have been arrested ; and while I am not sufficiently sanguine to expect his recovery, I think he will soon be able to return to his friends in the East. He gets up in the morning before breakfast time, walks about in the open air, and is looking forward to a complete recovery.

There are several points of interest in connection with this case, which have afforded me themes for thought. Among these, are :

1. The hæmoptyses. I had never before noticed this symptom in such rapidly advancing cases, and had been led to believe that it did not often occur. One eminent writer, Dr. James E. Pollock, in a lecture on this subject, published in the *London Medical Times and Gazette* for July 25th, 1874, says : "Acute tuberculosis has no hæmoptysis."

2. The occurrence of sudden, profuse hæmoptysis upon the receipt of a letter containing unpleasant news ; excited, he burst a blood-vessel.

3. The fact that many of these hæmoptyses, among them the first, occurred while the patient was lying quietly in bed, without any known exciting cause.

4. The occurrence of hæmoptyses without any previous fluctuation of temperature, or other prodrome.

5. The complete cessation of these hæmoptyses since the transfusion is noteworthy, in view of the statements of Tabouré,* who has had considerable experience with this operation. He gives it as his opinion, that the substitution of defibrinated for normal blood sets up a sort of hæmophilia.

6. The yielding of the night sweats as a result of the improved nutrition following the transfusion, while medicines had previously failed.

7. The difference in the number of respirations per minute, before and after the operation, from 34 to 22.

8. The non-occurrence of troublesome symptoms after the operation. No phlebitis or other untoward sequel occurred to either patient or donor. I had been prepared to expect rigors, an aggravation of the dyspnœa, hæmaturia, or some other troublesome symptom, but instead, bodily comfort and peace of mind immediately took the place of great mental and physical distress. How much of this is traceable to the fact that human blood was used instead of that of some other species of animal, I am unable to say; but bad results have more generally followed transfusion when blood from another species of animal has been used. Although the urine was not examined chemically or microscopically, no apparent change occurred in this secretion subsequent to the operation.

9. The simplicity of the operation. Performed in this way, it requires no special, complicated apparatus, nothing but a pair of scissors, or a knife, and a syringe. And as the hypodermic syringe is always at hand, transfusion is an easy matter. I am indebted to Surgeon W. C. Spencer, of the Army, for the idea of the system of ligatures, but I believe this is the first application of the idea. In this connection I cannot refrain from transcribing an expression of Druitt's, which occurs in his work on Surgery: "It is consolatory, therefore, to know, that most of the successful cases of transfusion have been performed with common pewter or brass syringes."

* *London Medical Times and Gazette*, Sept. 5, 1874.

10. The small quantity of blood used. Before two ounces had been injected, the patient became easier and more comfortable, the dyspnœa had nearly disappeared, the number of respirations per minute perceptibly lessened, and as soon as he expressed a "sense of fullness in the head," I desisted. I have not now the literature of the subject at my command, but I can remember instances in which eight to twelve and even sixteen ounces are spoken of as necessary.

11. The force necessary to introduce the blood. This, while it would not surprise a demonstrator of anatomy who had been accustomed to the injection of cadavers, was much more than I had supposed would be required to introduce a fluid into a living vein of the size of the median cephalic.

12. The use of defibrinated venous blood. Professor Albani is credited with having said that he does not think that any fluid obtained by defibrination is worthy the name of blood ;* and Gesellius, in his monograph, says defibrination is not only useless but injurious, because small fibrinous clots and rouleaux of blood corpuscles are always abundantly present in blood which has been whipped and stirred to remove its fibrin, and there is, therefore, the danger of the production of embolisms by their means—and further, that defibrinated blood is to a certain extent dead, because its corpuscles have become exhausted in the production of the fibrin, and the transfused blood will be less and less beneficial the more perfectly the defibrinating process has been carried out.†

Despite the caution which has been often given to avoid using, as a donor, a person addicted to liquor, I used blood from a man, who, in his enthusiasm, had nearly tested his capacity for liquor just before the operation ; but the quality of the blood seems not to have been materially injured.

* *London Medical Times and Gazette*, Sept. 5, 1874.

† *London* " " " Dec. 19, 1874.

To Dr. C. V. Petteys, A. A. Surgeon, U. S. A., and Dr. J. L. Mills, Medical officer to the Spotted Tail Indian Agency, great credit is due for their assistance and counsel in this case.

Feb. 29, 1876. The patient weighed 114 pounds to-day, and is increasing in weight at the rate of three-quarters of a pound daily.

CAMP SHERIDAN, Neb., Feb. 29, 1876.

## Figures and Tables

**Figure f1:**